# Oppression-Based Stress and Alcohol Inequities Among Sexual and Gender Minority People: An Intersectional Multilevel Framework

**DOI:** 10.35946/arcr.v44.1.05

**Published:** 2024-09-05

**Authors:** Ethan H. Mereish

**Affiliations:** Lavender Lab, Department of Psychology, University of Maryland, College Park, Maryland

**Keywords:** alcohol, sexual minority, gender minority, stress, oppression, intersectionality, motives, social norms

## Abstract

**PURPOSE:**

Sexual and gender minority (SGM) people are at heightened risk for alcohol use, hazardous drinking, and alcohol use disorder compared to heterosexual and cisgender individuals. This paper: (a) presents an oppression framework that integrates intersectionality, stress, stigma, and addiction-based theories to examine the complex and nuanced ways oppression-based stress (e.g., minority stress) leads to sexual orientation and gender identity inequities in alcohol use; (b) conducts a narrative review that summarizes recent and novel advancements in the literature on the impact of oppression-based stressors on alcohol use outcomes across structural, interpersonal, and intrapersonal domains among SGM people; and (c) provides future research and intervention directions for the alcohol field.

**SEARCH METHODS:**

A select review of the literature was conducted on July 10, 2023, using multiple electronic databases (i.e., PsycInfo, PubMed, Web of Science) and focusing on studies that had examined the associations between oppression-based stressors and alcohol use outcomes across structural, interpersonal, and intrapersonal levels. Search terms focused on alcohol consumption; SGM people, particularly SGM people of color; and oppression-based stress. Cross-sectional studies that focused on heterosexism-based and anti-bisexual oppression-based stressors at the interpersonal or intrapersonal levels and alcohol use outcomes were excluded as they have been included in prior reviews of the literature.

**SEARCH RESULTS:**

The initial and combined search across the databases resulted in 3,205 articles. Of those, the narrative review included 50 peer-reviewed articles that focused on the following four areas of the literature on the associations between oppression-based stressors and alcohol use outcomes: (1) experimental, longitudinal, and experience sampling studies of heterosexism- and anti-bisexual oppression-based stressors (22 articles); (2) any studies of cissexism-based stressors (12 articles); (3) any studies of intersectional oppression among SGM people of color (seven articles, one article overlapped with the first category and another overlapped with the fourth category); and (4) any studies of structural oppression (11 articles).

**DISCUSSION AND CONCLUSIONS:**

Results of this narrative review indicate that mounting evidence implicates oppression-based stress in inequities in alcohol use, hazardous drinking, and alcohol use disorder in SGM populations. This reflects SGM people’s embodiment of oppression and injustice at the structural, interpersonal, and intrapersonal levels. Given some inconsistent and mixed patterns of findings, future research needs greater specificity in drinking inclusion criteria, robust and well-validated measures, more attention to culturally and developmentally relevant moderating and mediating mechanisms across the lifespan, application of sophisticated methodologies, and integration of intersectional and addiction frameworks.

Sexual minority (e.g., lesbian, gay, bisexual, queer, asexual) and gender minority (e.g., transgender, nonbinary, other gender-diverse) people are at heightened risk for alcohol use, hazardous drinking,* and alcohol use disorder (AUD) compared to their heterosexual and cisgender counterparts.^2^–^6^ (For definitions of various sexual and gender minority [SGM] populations, see, for example, the National Institutes of Health Office of Diversity, Equity, and Inclusion website.^7^) Alcoholdisparities are concerning, given their co-occurrence with and bidirectional impact on myriad mental health outcomes (e.g., mood and anxiety disorders) and physical health outcomes (e.g., cardiovascular disease, sexually transmitted infections) and related disparities.^8^–^12^ Health disparities based on sexual orientation and gender identity are considered health inequities because they are rooted in historical and societal systems of oppression (e.g., heterosexism, anti-bisexual prejudice, cissexism),^13^,^14^ and are avoidable health outcomes that reflect socially unjust conditions.

Sexual orientation inequities in alcohol outcomes have been documented across the alcohol use and addiction continuum. Epidemiological work indicates that sexual minority youth initiate alcohol use at younger ages than their heterosexual peers,^15^–^18^ and alcohol use persists (and sometimes accelerates) into adulthood.^16^,^18^–^23^ Sexual minority people—girls and women in particular—are more likely than heterosexual individuals to engage in hazardous drinking, such as binge or heavy episodic drinking (HED; defined, unless otherwise indicated, as four or more drinks within a 2-hour period for females or five or more drinks within 2 hours for males^24^) and high-intensity HED (i.e., drinking twice or more times the threshold for HED^24^),^25^–^28^ to experience negative alcohol-related consequences and harms,^29^,^30^ and to engage in polysubstance use.^31^ Sexual minority individuals also have a higher prevalence of AUD than do heterosexual people.^32^–^35^ While there has been a decline in alcohol use among young people in the United States, sexual orientation inequities remain stable and have widened for sexual minority girls and women.^28^,^36^–^38^ Representing the heterogeneity that exists within the sexual minority community, mounting evidence from representative samples consistently demonstrates that sexual minority girls and women as well as plurisexual (e.g., bisexual, pansexual, queer) individuals are at greatest risk for alcohol use, hazardous drinking, and AUD, whereas these inequities are evident but sometimes less consistent for sexual minority boys and men.^5^,^17^,^25^–^27^,^39^–^44^

Gender identity inequities in alcohol consumption have been documented across multiple alcohol outcomes. In alcohol and other substance use initiation, gender identity inequities are evident as early as 12 years of age, and these disparities widen over adolescence.^18^ Representative and nonprobability studies indicate that gender minority people are more likely to report alcohol and other substance use, HED, and polysubstance use than are their non-transgender or cisgender peers.^3^,^31^ Compared with cisgender adults, transgender adults have more negative consequences related to alcohol use,^45^ including alcohol-related sexual assault and suicide ideation.^46^–^48^ While some population-level studies do not document alcohol inequities,^49^–^52^ some of these studies were limited in their sample characteristics (e.g., clinical samples of treatment-seeking participants)^49^,^50^ and measurement of alcohol use (e.g., poor scale reliability).^51^ Additionally, results from multiple large-scale studies indicate that transgender adults are more likely than cisgender adults to have a diagnosis of AUD and other substance use disorders.^50^,^53^–^55^ Gender minority adults are more likely than cisgender adults to report that they wanted help to reduce their use of alcohol and other substances.^56^

Transgender and other gender minority people, including binary (e.g., transgender women and men) and nonbinary (e.g., genderqueer, agender) individuals,^57^ have differing alcohol use patterns. Transfeminine people have higher prevalence of AUD and other substance use disorders compared to transmasculine people.^53^ Transgender people who identify as a sexual minority^58^,^59^ and who are assigned female at birth may be at heightened risk for alcohol use outcomes.^48^,^60^ Nonbinary people may be at greater risk for alcohol use, hazardous drinking, AUD, and substance use disorders compared to other transgender people^51^,^61^–^63^ and cisgender women.^63^,^64^ However, some studies show less alcohol use for nonbinary individuals compared to cisgender women,^47^,^65^,^66^ and one study found no differences in alcohol use between nonbinary and binary transgender youth.^67^ As such, more research is needed to understand the unique drinking patterns of nonbinary and other gender-diverse individuals and how they compare to cisgender and other gender minority groups.

## Alcohol Inequities at the Intersection of Race/Ethnicity

An emerging literature base shows that alcohol inequities based on sexual orientation and gender identity vary at the intersection of race and ethnicity. The somewhat consistent pattern in the extant literature is that such inequities exist between Black, Indigenous, and People of Color (BIPOC) SGM individuals and their heterosexual peers of the same race/ethnicity. Other work has examined racial differences in alcohol use outcomes within SGM groups, and patterns of findings are less clear and vary by age group. These disparate findings may partially be explained by limitations of study designs. Some studies aggregated racial and ethnic groups into one BIPOC group, which ignores known racial and ethnic differences in drinking patterns as well as differences in the long-term effects of drinking (e.g., racial differences in AUD or alcohol-related consequences but not quantity or frequency of alcohol use). Additionally, other studies aggregated across adulthood without considering developmental changes in drinking across the lifespan.

Findings from nationally representative probability studies (i.e., studies that involve a randomly selected sample representing the population) and nonprobability studies (i.e., studies that use nonrandom samples, such as convenience or community samples) show that SGM BIPOC youth report greater alcohol and polysubstance use compared to their heterosexual and cisgender peers of the same race/ethnicity;^68^–^72^ these inequities are especially elevated between Hispanic/Latine and Black American transgender students and their same-race peers.^72^ In contrast, racial/ethnic differences in alcohol use among SGM youth show less consistent patterns. A large study of bisexual youth found that Black bisexual girls were less likely to engage in HED compared to White bisexual girls, whereas Black and White bisexual boys and Hispanic and White bisexual youth had comparable levels of HED.^73^ A longitudinal study found that Black SGM youth reported less alcohol use than all other racial groups of SGM youth in the sample.^74^ Other work indicates that Black young sexual minority men had lower increases in alcohol use from adolescence to adulthood compared to their White counterparts, whereas Hispanic/Latine young sexual minority men had higher increases in their alcohol use trajectories from adolescence to adulthood compared to their White counterparts.^75^ A recent study found that Latine and Black gender minority youth had the highest prevalence of recent alcohol use, HED, and cannabis use compared to most other SGM youth; Asian/Pacific Islander SGM youth had some of the lowest prevalence rates of alcohol use compared to other SGM groups.^76^

The patterns of findings for racial/ethnic differences in sexual orientation inequities in alcohol use among adult samples are gendered. Findings from probability samples show that Black and Hispanic/Latine sexual minority women are at greater risk for multiple alcohol use outcomes (e.g., alcohol quantity and frequency, HED) and other substance use compared to same-race peers and, to some extent, to White heterosexual and sexual minority women.^44^,^77^–^81^ With the exception of two studies,^82^,^83^ these findings are also present in nonprobability samples of sexual minority women.^84^,^85^ Conversely, several probability and nonprobability samples have found no differences between BIPOC sexual minority men and BIPOC heterosexual men in HED,^39^,^44^,^80^,^86^ heavy drinking (e.g., frequent and high-volume daily, weekly, or episodic drinking),^39^,^81^,^86^ alcohol-related consequences,^39^ and substance use problems,^84^ whereas other probability studies indicated lower heavy weekly drinking (defined as more than 14 drinks per week) and HED for Black sexual minority men compared to Black and White heterosexual men.^86^ In contrast, other work found that Black, Hispanic/Latine, and White sexual minority men were more likely than same-race heterosexual men to be current drinkers.^81^

Research examining racial/ethnic differences in gender identity disparities in alcohol use is scant and has inconsistent patterns.^3^ A large-scale study indicated that Hispanic/Latine transgender adults had a greater likelihood of current alcohol use and engagement in HED compared to non-Hispanic/Latine White transgender adults.^66^ This study also found that Black and “other” BIPOC transgender adults had a lower likelihood of current alcohol use and frequency of HED compared to White transgender adults, and no differences were found between Biracial/Multiracial and White participants.^66^ Two community studies found no racial/ethnic differences in past-year alcohol dependence (as defined by the *Diagnostic and Statistical Manual of Mental Disorders*, 4th Edition) among transgender women^87^ and in drinking five or more alcoholic drinks on one occasion in the past year among Canadian transgender people.^88^ A study of college students found that BIPOC transgender college students were at greater risk than White transgender college students for alcohol-related blackouts.^45^

## Oppression as a Unifying Framework

Building on prior established oppression theories and models, this paper focuses on oppression as an organizing framework that centers on multiple and intersecting types of oppression and the harms that they inflict on marginalized people. Oppression involves an unjust system of power, privilege, and domination, wherein some social groups have more power and privilege than other groups, and they intentionally and unintentionally use this power and privilege to maintain domination over oppressed groups.^13^,^14^,^89^–^91^ Oppression is perpetrated explicitly and implicitly at multiple levels, including structural (i.e., systemic, cultural, and institutional) and individual (i.e., interpersonal and intrapersonal) levels.^13^,^14^,^89^–^92^ It inflicts stress (i.e., oppression-based stress) at these levels and results in deleterious consequences and harms for oppressed social groups (e.g., SGM people). Oppression threatens the social safety of marginalized individuals and creates processes that reinforce an absence of safety.^93^ Oppression is also a shaming process that further silences and isolates oppressed individuals to enable oppressors to maintain power.^94^ Moreover, oppression is both a process (i.e., historical or ongoing process of oppression of marginalized groups through restriction, dehumanization, and deprivation) and a state (i.e., state of inequality).^89^ Similarly, marginalized individuals are both subjected to the traumatic process of oppression (i.e., oppression-based stressors) across multiple levels and experience oppression-based stress as a state that results from direct or vicarious exposure to oppression-based stressors. Due to its sociohistorical roots, oppression-based stress and related trauma can be transmitted across generations.^90^

Utilizing an oppression framework is inclusive of and integrates multiple well-established but often disparate theoretical models that focus on discrimination or stigma to explain health inequities among varying marginalized groups (e.g., minority stress and sexual stigma models for SGM people, racism-based stress for BIPOC, sexism models for women).^13^,^14^,^90^,^92^,^95^–^100^ A major limitation of some of these theoretical models is their focus on only one axis of oppression (e.g., heterosexism, cissexism, racism, sexism) and their over-focus on individual-level processes (e.g., discrimination). For example, the primary guiding theoretical model for understanding health inequities among SGM people is the minority stress model, which initially focused on heterosexism-based stress at the interpersonal and intrapersonal levels (i.e., minority stress) among sexual minority people and later adapted to consider cissexism-based stress and its negative impact on gender minority people.^96^,^97^,^101^

Intersectionality theory, which was developed by Black feminist scholars, posits that social groups concurrently experience multiple oppression-based stressors (e.g., racism, sexism, classism, heterosexism, anti-bisexual prejudice, cissexism, ableism, ageism, xenophobia, colonialism, antisemitism, Islamophobia, weight-based oppression) related to their intersecting and marginalized social positions and identities (e.g., race, ethnicity, gender, class, sexual orientation, religion). These multiple forms of oppression-based stressors are perpetuated and concurrently reinforced by interconnected structural systems of oppression.^102^–^105^ Moreover, identity is not an additive sum or multiplicative of several social positions or oppressions (i.e., experiences of SGM BIPOC individuals are not simply the sum of all of their oppressions); in fact, social identities are concurrently influencing each other and are shaped by a matrix of power, privilege, and oppression.^104^,^106^ This notion is consistent with the mixed findings in alcohol inequities at the intersection of sexual orientation, gender, race, and ethnicity, wherein some marginalized groups do not have higher risk for alcohol use compared to privileged groups.^84^

An oppression framework reflects prior oppression and intersectional approaches that underscore that each individual is within a context of interlocking systems that create and perpetuate health inequities; this framework helps shifts the lens from the individual to the systems and structures of oppression.^91^,^102^,^103^,^106^–^108^ This is important as the over-focus on the individual and their coping and resilience capabilities can be pathologizing and disempowering and can further perpetuate the status quo of oppression.^91^,^102^,^103^,^106^–^108^ Moreover, centering the focus on oppression allows for an intersectional understanding of the common processes that exist across all and intersecting forms of oppression (e.g., power, privilege, domination, subordination) and their negative impacts on oppressed groups.^91^,^103^ In doing so, disrupting and dismantling systems of oppression are an integral part of promoting and achieving health equity.

## Oppression-Based Stress Among SGM People

SGM individuals experience multiple systems of oppression, including heterosexism (i.e., oppression of sexual minority people), anti-bisexual oppression (i.e., oppression of bisexual and other plurisexual people), and cissexism (i.e., oppression of transgender and nonbinary people) at multiple levels that are linked to and shape each other. For example, structural oppression can perpetuate interpersonal oppression (e.g., higher rates of hate crimes and harassment of SGM people) and intrapersonal oppression (e.g., internalized heterosexism or cissexism).

### Structural Oppression

Structural oppression, also known as structural, institutional, or cultural stigma, includes heterosexist and cissexist policies or laws and cultural norms or attitudes that create or reinforce inequities among SGM groups to maintain power and privilege among heterosexual and cisgender groups.^13^,^14^,^92^,^109^,^110^ Despite increased visibility and positive changes for SGM individuals in the United States over the past two decades, structural oppression (e.g., cissexist, heterosexist, racist, and sexist legislation) and violence toward SGM people have increased significantly.^111^

Structural heterosexism and cissexism create structural-level barriers and stressors and exacerbate other social determinants of health. For instance, SGM people are more likely than heterosexual and cisgender individuals to encounter structural barriers, such as lack of access to care and health insurance,^112^,^113^ and to experience other inequities in social determinants of health, such as housing insecurity and instability, food insecurity, and economic hardship (e.g., unemployment).^52^,^112^–^115^

Furthermore, structural oppression operates overtly and covertly by shaping cultural and social norms, which are well-known factors that impact alcohol use.^116^ Among SGM communities, oppression shapes drinking and other substance-related social norms through exploitative economic and capitalistic factors. Due to historic and systemic heterosexism and cissexism, there is a lack of safe and affirming spaces for SGM people to be in community with each other.^2^,^117^ SGM bars, which often were one of the few spaces for SGM people to connect and resist multiple forms of oppression,^2^,^117^ frequently involved alcohol and other substance use. Moreover, economic forces have targeted and exploited SGM communities and organizations. Alcohol companies use marketing strategies (e.g., sponsorship of events, advertising) to target specific communities, which ultimately influences their drinking norms and their culture more broadly.^116^,^118^ Scholars have indicated that alcohol and tobacco companies have engaged in targeted marketing of SGM communities.^119^–^122^ As such, SGM culture has become intertwined with alcohol and other substance use; in fact, sexual minority individuals perceive drinking as a normed component of queer identity and culture.^123^ This has influences on SGM people’s social networks,^124^,^125^ permissive drinking norms,^126^ misperceptions of others’ drinking,^127^,^128^ and lower perceived risks associated with drinking.^129^

### Interpersonal and Intrapersonal Oppression

Oppression-based stressors at the interpersonal level, commonly known as distal minority stressors in the minority stress model,^96^,^97^,^101^ include heterosexist, anti-bisexual, or cissexist violence, discrimination, harassment, microaggressions, non-affirmation (e.g., being misgendered), body- or gender-policing, and transitioning-identity stress.^96^,^130^,^131^ These stressors can be directly or vicariously experienced in every domain of SGM people’s lives,^130^,^132^,^133^ are quite pervasive, inflict shame and trauma, impact experiences of social safety, and may be experienced from within the SGM community (e.g., bisexual prejudice, cissexism, racism, sexism, and/or ableism from within the SGM community; competition or social exclusion for not fitting rigid expectations related to success, sex, status, or body image).^93^,^96^,^134^–^136^ Furthermore, SGM individuals who are in romantic relationships can experience couple-level oppression-based stressors,^137^ and their own and their partner’s oppression-based stressors can have crossover effects in the relationship.^138^,^139^ Moreover, SGM individuals, especially sexual minority women, plurisexual women, and gender minority individuals, are disproportionally more likely to experience general life and traumatic stressors (e.g., verbal, physical, and sexual victimization) over their lifespan than are heterosexual and cisgender individuals.^140^–^143^

At the intrapersonal or internalized level, oppression-based stressors reflect processes related to the internalization of oppression, which are considered proximal minority stressors and self-stigma in the minority stress model and sexual stigma theory, respectively. Examples include internalized oppression (e.g., internalized heterosexism, anti-bisexual prejudice, and/or cissexism, which reflect internalized shame); felt or awareness of oppression; anticipation, vigilance, or expectation of oppression-based stressors; sexual and/or gender identity concealment;^92^,^96^,^97^,^101^ and gender dysphoria.^144^ It is important to note that intrapersonal oppression-based stressors manifest in both interpersonal and intrapersonal domains and are not solely an intrapersonal process; they often manifest in an interaction between individuals and their oppressive environment. Additionally, oppression at the structural and interpersonal levels instills, shapes, and reinforces intrapersonal oppression-based processes.

In addition to oppression-based stressors that impact SGM people more broadly, SGM BIPOC experience unique and intersectional forms of oppression-based stressors at the intrapersonal and interpersonal levels. Documented forms of intersectional oppression-based stressors that SGM BIPOC uniquely experience are racism and invisibility in SGM communities, heterosexism, cissexism, anti-plurisexual oppression in their own racial and ethnic communities, and racism in dating and close relationships.^105^,^145^ They also experience internalized racism as well as other forms of racism-based stressors that BIPOC are regularly exposed to over their lifetimes.^13^,^14^,^90^,^98^–^100^

## Oppression and Drinking Motivations Among SGM People

Several reviews of the SGM alcohol literature highlight that oppression-based stressors are a risk factor for alcohol use and hazardous drinking among SGM individuals.^2^,^4^,^5^,^40^,^146^–^151^ For example, a meta-analysis of alcohol and substance use among sexual minority youth found that oppression-based stressors were some of the strongest risk factors for substance use.^151^ Oppression-based stressors lead to poor health outcomes, including hazardous drinking, in part due to their disruptive and deleterious impacts on cognitive, affective, behavioral, physiological, and interpersonal and relational processes.^94^,^152^–^154^ Addiction models can help further explain how oppression-based stressors lead to drinking behaviors and other alcohol use outcomes among SGM people. Congruent with negative reinforcement models,^155^ the self-medication hypothesis,^156^ and motivational models of substance use,^157^,^158^ SGM people may use alcohol to cope with, regulate, or self-medicate negative emotions (e.g., distress, shame, hopelessness, anger) that are instigated by oppression-based stressors.

Motivational models of addiction indicate that individuals have varying motivations for alcohol consumption and these motives impact their drinking behaviors.^157^,^158^ Consistent with oppression-based stress and negative reinforcement models, SGM people may drink to cope with distress instigated by general and oppression-based stressors or to enhance their mood or affective states that may be dampened by stress. In fact, meta-analytic work indicates that coping motives are one of the strongest predictors of drinking problems among the broader literature.^159^ Qualitative work has found that sexual minority women and Black and Latine sexual minority adults use alcohol to cope with heterosexism-based stressors^123^,^160^–^162^ and intersectional oppression-based stressors.^123^ Similarly, researchers found that about one-fourth of transgender adults used alcohol or drugs to cope with cissexist discrimination,^163^ and transgender college students were more likely than their cisgender peers to endorse coping and mood enhancement drinking motives.^45^ Coping motives partially mediate the associations between heterosexism- and cissexism-based stressors and alcohol use and consequences among SGM adults.^164^–^169^

Other drinking motives that influence SGM’s alcohol use also may be influenced by oppression. In alignment with social learning theory,^170^ social and conformity motives are relevant as SGM people may be motivated to drink due to peer modeling of substance use and permissive drinking social norms that are shaped by alcohol companies targeting SGM communities. Moreover, sexual enhancement drinking motives may be important as SGM individuals may drink to initiate or enhance their sexual experiences,^117^,^161^,^162^,^171^ which have been systematically stigmatized. Lastly, SGM individuals may be motivated to drink to challenge the rigid binary gender socialization, engage freely in gender expression, and affirm their gender identities,^117^,^172^ which have been policed by oppression.

## Purpose of Present Narrative Review

As described above, alcohol inequities that SGM people experience have been widely explained by theoretical models of oppression, stigma, and stress. The purpose of this paper is to review and summarize recent developments and advancements in the literature that examine the impact of oppression-based stressors on alcohol use outcomes in SGM populations across multiple levels (e.g., intrapersonal, interpersonal, and structural). A common finding of prior SGM alcohol reviews has been that the field has largely used cross-sectional designs and has focused primarily on heterosexism-based stressors among sexual minority people.^2^,^4^,^5^,^40^,^146^–^151^ Thus, this paper focuses only on methodologies that move beyond cross-sectional designs (i.e., experimental, longitudinal, and experience sampling) to understand the associations between heterosexism-based and anti-bisexual oppression-based stressors among SGM people. Previous SGM alcohol reviews have primarily focused on sexual minority people and have not considered other forms of oppression (e.g., cissexism, intersectional oppression), limiting our understanding of the literature on cissexism- and intersectional oppression-based stressors and alcohol use outcomes among SGM people and especially SGM BIPOC. Therefore, a second aim of this review is to summarize the existing literature on cissexism-based stressors and alcohol use outcomes among gender minority people as well as the literature on intersectional oppression among SGM BIPOC. Given that oppression manifests at the structural level and the limited attention of prior work on this type of oppression, the third aim of this paper is to review the literature on structural heterosexism and cissexism and alcohol use outcomes among SGM people. All study designs, including cross-sectional studies, were considered for the second and third aims.

## Search Methods Employed

A select review of the literature was conducted on July 10, 2023, using multiple electronic databases (i.e., PsycInfo, PubMed, Web of Science). Search terms focused on alcohol consumption (e.g., alcohol, drink*); sexual minorities (e.g., LGBT, lesbian, gay, bisexual); gender minorities (e.g., transgender, nonbinary, gender diverse); and other relevant areas, such as intersectionality, oppression, minority stress, racism, and BIPOC individuals (e.g., intersectionality, minority stress, discrimination, stress, structural stigma, heterosexism, cissexism, racism, people of color, racial minorities). These searches captured 3,205 articles. Duplicates were removed. Each article’s title and abstract were reviewed, and full-text articles that were of relevance to this paper were retrieved and reviewed in detail. Cross-sectional studies that focused on heterosexism and anti-bisexual oppression-based stressors at the interpersonal or intrapersonal levels and alcohol use outcomes were excluded as they have been included in prior reviews of the literature.

## Results of the Literature Search

As a result of the search, this review included 50 articles that focused on the following four areas of the literature examining the associations between oppression-based stressors and alcohol use outcomes: (1) experimental, longitudinal, and experience sampling studies of heterosexism and anti-bisexual oppression-based stress (22 articles); (2) any studies of cissexism-based stress (12 articles); (3) any studies of intersectional oppression among SGM BIPOC (seven articles, which included one article that overlapped with and is also included in the first category and one article that overlapped with and is also included in the fourth category); and (4) any studies of structural oppression (11 articles).

## Results of the Reviewed Studies

### Heterosexism and Anti-Bisexual Oppression-Based Stress

One development in the literature base is the use of experimental research. Despite the utility of experimental methodologies, to date only one study has examined the impact of heterosexism-based stress on alcohol outcomes among sexual minority individuals in a controlled laboratory setting. Researchers developed a novel experimental paradigm to assess heterosexism-based stress, which included mood induction using picture stimuli to manipulate heterosexism-based stress. This study provided the first experimental evidence to date that vicarious exposure to heterosexism elicited negative affect and alcohol craving as well as enhanced alcohol cue reactivity effects among sexual minority young adults who engaged in hazardous drinking.^133^ This study also found that, compared with a neutral condition, elevated heterosexism-based psychophysiological stress reactivity, as assessed by the startle response, was correlated with more recent alcohol use.^173^

Another major advancement and emerging literature base leverages longitudinal and intensive longitudinal/experience sampling (e.g., ecological momentary assessments, daily diary studies) designs to understand the concurrent and prospective within-person associations between oppression-based stressors and alcohol outcomes over time and in the natural environment. Among longitudinal studies with extended assessments periods (e.g., months), heterosexism-based stress was associated with concurrent and prospective alcohol use outcomes; however, findings were inconsistent. Four longitudinal studies ranging from three to seven waves of assessments over nine months to 2.5 years among SGM young adults found concurrent but not prospective positive associations between heterosexism-based stressors (e.g., microaggressions, victimization, internalized heterosexism) and several drinking outcomes.^74^,^165^,^174^,^175^ One of these studies, however, found neither concurrent nor prospective associations between internalized heterosexism and alcohol use.^175^ In contrast, five other longitudinal studies documented prospective associations between heterosexism-based stressors and alcohol use outcomes (e.g., HED, alcohol-related consequences) for sexual minority adolescents,^124^ girls,^176^ and college students,^167^ as well as young sexual minority women.^177^,^178^

Daily diary studies using 14- to 28-day monitoring periods with one survey per day among sexual minority women demonstrated some mixed findings regarding the within-person associations between heterosexism-based stress and same-day and next-day drinking outcomes. Studies provided support for the daily associations between interpersonal heterosexism-based stressors (e.g., discrimination, microaggressions) and same-day alcohol use,^138^,^179^ same-day negative alcohol-related consequences,^180^,^181^ and next-day quantity of drinks and HED^179^ among young sexual minority women.^138^,^179^–^181^ A dyadic analysis of the same dataset of sexual minority women^179^ found that heterosexism-based stressors of a sexual minority woman’s romantic partner were associated with her own same-day HED.^138^ These studies also found nonsignificant associations between oppression-based stressors and the assessed alcohol outcomes for same- and next-day alcohol use. Only one study assessed intrapersonal heterosexism-based stress (i.e., sexual identity concealment) and found that it was not associated with any of the assessed alcohol outcomes.^179^ Some of the mixed findings might be explained by the measures of oppression-based stressors used, as some only used one item to assess this construct,^181^ and by mediating mechanisms, such as the ones found in one study to explain their associations (e.g., coping efficacy, social anxiety).^181^

Daily diary studies of stress and drinking among sexual minority cisgender men provide less consistent patterns but highlight potential moderating variables (e.g., race and structural oppression). In one daily diary study, stressful daily events were associated with same-day drinking among heavy-drinking (defined as an average weekly consumption of at least 24 standard drinks over the past 90 days) sexual minority cisgender men who were treatment seeking; however, this study did not explicitly assess oppression-based stressors.^182^ In contrast, another study found that heterosexism-based stressors were not associated with same-day or week-level drinking quantity or heavy drinking (defined as drinking five or more drinks in one sitting) in sexual minority cisgender men.^183^ However, this study found that heterosexism stressors were associated with less same-day drinking for BIPOC participants, but were not associated with drinking frequency for White participants.^183^ Another daily diary study of sexual minority men found that gay-related rejection sensitivity was not significantly associated with same-day alcohol use; however, it was associated with greater alcohol use among sexual minority men living in states with greater structural heterosexism (i.e., assessed presence of state-level heterosexist policies and social attitudes) than in states with less structural heterosexism.^184^

Three ecological momentary assessment studies provided more nuanced findings for the within-day associations between heterosexism-based stressors and alcohol outcomes. One study indicated that momentary heterosexism/cissexism-based stressors (e.g., discrimination) were associated with concurrent and later substance use on the same day among SGM young adults.^185^ However, this study did not discern these effects for alcohol use specifically. Another study found that parental heterosexism (e.g., rejection) assessed at baseline was associated with greater momentary alcohol and cannabis craving and negative affect among sexual minority youth who used nicotine;^186^ the same study also found momentary associations between heterosexism stressors and nicotine craving.^187^ The third study found that within-day associations between heterosexism stressors and alcohol use were moderated by baseline alcohol use frequency and scores on the Alcohol Use Disorders Identification Test (AUDIT), wherein heterosexism stressors were associated with alcohol use only among SGM young women who reported more frequent alcohol use and those who had higher AUDIT scores at baseline compared to participants with less frequent alcohol use or lower AUDIT scores.^188^

### Cissexism Stress and Alcohol Use Among Gender Minorities

Several studies have documented that multiple forms of cissexism-based stressors at the interpersonal level are associated with alcohol use, hazardous drinking, other substance use, and substance use disorder among gender minority individuals;^3^,^66^,^147^,^189^–^192^ however, the results have been inconsistent, and studies relied primarily on cross-sectional designs.

Two cross-sectional studies found that victimization (i.e., bullying and harassment) was associated with greater alcohol and other substance use^191^ and HED^193^ among gender minority youth; moreover, victimization mediated alcohol inequities between gender minority and cisgender youth.^191^,^193^ Two other studies of gender minority adults found that cissexism-based discrimination, especially in specific contexts (i.e., public spaces, housing), was associated with more alcohol use, engagement and frequency of recent HED, and alcohol-related problems.^66^,^190^ In contrast, other cross-sectional studies showed that cissexism stressors were not associated with consuming five or more drinks on one occasion in the past year^88^ or with consuming four or more drinks in a single day within the past three months^194^ in gender minority adults. In a 3-year longitudinal study, cissexism-based verbal and physical abuse was associated with drinking five or more drinks in one sitting and other substance use in transgender women; these effects were also mediated by depressive symptoms.^195^

Limited studies have assessed the effects of cissexism-based stress at the intrapersonal level. In one cross-sectional study, several cissexism-based stressors (i.e., internalized stigma, gender identity concealment, gender dysphoria, and anticipated stigma) were individually and together associated with problematic alcohol use in an online sample of transgender and nonbinary adults, and these associations were mediated through drinking-to-cope motives.^169^ Similarly, other work found that gender dysphoria was associated with greater heavy drinking.^194^ Another study found that expectations of oppression were positively correlated with hazardous drinking among a sample of gender minority people; however, this association was not significant in multivariate models.^196^

### Intersectional Oppression and Alcohol Use Among SGM BIPOC

A small but burgeoning body of research has focused on multiple forms of oppression and drinking behaviors among SGM BIPOC. In a longitudinal study of young Black and Latine sexual minority men, researchers identified several groups based on their experiences of oppression-based stressors, including individuals who experienced minimal oppression (i.e., infrequent heterosexism and racism stressors), individuals who primarily experienced multiple forms of the same single-axis of oppression (i.e., heterosexism or racism), and a group who experienced compound oppressions (i.e., frequent heterosexism as well as racism stressors).^197^ Compared to the minimal oppression group, the other groups had a higher likelihood of drinking, with the compound oppression group being at the highest risk.^197^ In sum, heterogenous experiences at multiple axes of oppression (i.e., heterosexism and racism) were associated with hazardous drinking.^197^ Similarly, in a longitudinal study of Black, Latine, and Multiracial sexual minority men, racism-based discrimination and gay rejection sensitivity were indirectly associated with heavy drinking (i.e., five or more drinks) over 12 months; this association was mediated through emotion dysregulation, such as depressive and anxiety symptoms.^198^ Other researchers found that racism and racist sexual objectification were associated with screening positive for AUD among Black and Latine/Latin American Canadian sexual minority men.^199^

Four studies examined the associations between intersectional oppression-based stress unique to SGM BIPOC (e.g., racism and invisibility in SGM communities, heterosexist and cissexist oppression in one’s own racial and ethnic communities, racism in dating experiences, racist sexual objectification) and alcohol use outcomes. These studies found that intersectional oppression-based stress at the interpersonal level was associated with recent and heavy alcohol use (i.e., five or more drinks in one sitting), hazardous drinking, alcohol-related consequences, and AUD in SGM BIPOC youth,^200^ Black and Latine sexual minority women and sexual minority men,^199^,^201^ and Latine sexual minority men.^202^

Only one study examined intersectional structural oppression by examining the intersection of structural heterosexism and racism; results indicated that the associations between structural racism and hazardous drinking were amplified for Black sexual minority men living in states with high structural heterosexism but not for their White counterparts.^203^

### Structural Oppression and Alcohol Use

A total of 11 studies focused on various domains of structural oppression and alcohol use. These studies assessed structural oppression in multiple ways, from measuring only one indicator (e.g., same-sex marriage laws) to indices of multiple indicators of structural oppression (e.g., laws that ban same-sex marriage or adoption, employment nondiscrimination laws, social attitudes toward sexual minority people). One study found that among sexual minority adults, living in states with high levels of structural heterosexism (e.g., banning same-sex marriage, negative attitudes towards sexual minority individuals) was associated with greater risk for AUD compared with living in states with lower levels of structural heterosexism.^204^ Other work found that the interaction of heterosexism at the structural and intrapersonal levels (i.e., gay-related rejection sensitivity) was associated with greater daily alcohol use among sexual minority men.^184^ In a study mentioned earlier, structural heterosexism and racism were independently associated with hazardous drinking among Black sexual minority men, but not White sexual minority men, and their interaction exacerbated this association.^203^ Moreover, structural heterosexism was related to the accessibility of SGM-inclusive and tailored programming at substance use treatment facilities.^205^

Some studies have examined the impact of SGM-protective laws and policies on drinking behaviors of SGM individuals. One study found that state-level transgender-inclusive and transgender-protective policies (i.e., an index of 35 different types of laws, such as laws related to nondiscrimination, parenting rights, health, safety, ability to correct listed gender on documents) were associated with less alcohol use, fewer poor mental health days, and more regular health care checks in transgender adults living in those states.^206^ Protective state-level sexual orientation policies also were associated with a lower likelihood of engaging in high-intensity drinking among sexual minority men, and, to some extent sexual minority women,^207^ and with less negative alcohol-related consequences for sexual minority women.^208^ Some of these protective polices were most beneficial in terms of drinking outcomes for Black and Latine sexual minority women, as well as sexual minority women with a high school education or less.^208^ Another study detected no disparities in HED between lesbian and heterosexual women in states with state-level protections, and disparities between bisexual and heterosexual women were lower in states with protections compared to states without these protections.^209^ Additionally, alcohol control-related policies significantly reduced HED for all women, but only in states that also had nondiscrimination laws and not in states without nondiscrimination laws.^209^ However, these findings were not documented for sexual minority men.^209^ In contrast to these findings, another study found that SGM equity laws (e.g., nondiscrimination laws) were associated with less cigarette use but more alcohol use and HED among SGM youth.^210^ Lastly, research on school-based protective structural factors—such as SGM-affirming organizations (e.g., Gender and Sexuality Alliances; GSAs) and anti-heterosexist bullying policies—found that the presence of such factors was associated with less alcohol use, HED, and other substance use for SGM youth compared to SGM youth who attended schools or lived in jurisdictions without such protective factors.^211^–^213^

## Discussion

Results of this narrative review indicate mounting evidence that implicates oppression-based stressors in inequities in alcohol use, hazardous drinking, and AUD in SGM populations. This reflects SGM people’s embodiment of oppression and injustice at the structural, interpersonal, and intrapersonal levels.^13^ Among the reviewed literature on heterosexism and anti-bisexual oppression, several studies have provided support for longitudinal and daily concurrent and prospective associations between oppression-based stressors and drinking outcomes; however, there are several mixed and inconsistent findings. With respect to cissexism, several studies have documented that multiple forms of cissexism-based stress at the interpersonal and intrapersonal levels are associated with alcohol use and hazardous drinking among gender minority individuals. Again, some of these findings have been inconsistent, and most studies relied primarily on cross-sectional designs. Among the intersectional oppression literature focused on the intersection of racism and heterosexism/cissexism, recent work showed that heterosexism and racism-based stressors at the interpersonal and structural levels were independently associated with alcohol use outcomes, and their intersection at the structural level exacerbated their independent structural effects on drinking; however, these associations to date have only been tested among BIPOC sexual minority men. Additionally, there is support for the associations between unique and intersectional oppression-based stressors and greater alcohol use outcomes among SGM BIPOC. Lastly, the structural oppression alcohol literature has yielded emerging and somewhat consistent evidence among SGM youth and adults that structural oppression is associated with greater alcohol use outcomes and that structural measures that protect SGM people are associated with less alcohol use. There is some potential evidence for gender differences in these associations. Given some inconsistent and mixed patterns of findings across the reviewed literature bases, future research needs greater specificity, robust and well-validated measures, and more attention to intersectional and addiction frameworks.

### Limitations and Gaps: Future Research Directions

Several weaknesses and much heterogeneity in the reviewed studies’ drinking inclusion criteria and assessment of different types of oppression-based stressors in the SGM populations may contribute to inconsistent patterns of results. First, the majority of cross-sectional and longitudinal studies, as well as some of the experience sampling studies, did not have alcohol use as an inclusion criterion. As such, study samples included SGM individuals who had never initiated or had abstained from alcohol use as well as individuals who used alcohol and had a wide range of drinking patterns (i.e., experimental, infrequent, occasional, frequent, and heavy use). This obscures understanding of the associations between oppression-based stressors and alcohol outcomes. Second, different types of oppression-based stress have received different levels of attention. Overall, there is a large focus on oppression-based stress at the interpersonal and intrapersonal levels and limited investigation of oppression at the structural level, despite the significant increase of anti-SGM legislation in the United States. Applying an oppression framework and examining the intersection of different forms of oppression at multiple levels can refine understanding of oppression-based stress and alcohol use and further clarify mixed and inconsistent findings. Additionally, more studies have focused on oppression-based stressors at the interpersonal level than at the intrapersonal level. There are mixed findings regarding the associations between internalized oppression, concealment, and alcohol use; as such, continued examination of internalized stigma and multiple other oppression-based stressors at the intrapersonal level (e.g., anticipation of oppression, gender dysphoria) is needed in advancing the field. Third, except for two measures developed to assess oppression-based stressors,^132^,^214^ experience sampling studies lacked well-validated measures of oppression-based stressors at the interpersonal and intrapersonal levels. Using validated measures can improve research rigor and allow for comparisons across studies. Given the diverse and unique ways oppression-based stressors can be experienced as well as the recent identification of additional and different types of heterosexism- and cissexism-based stress (e.g., non-affirmation, body or gender policing, couple-based stress, anti-bisexual prejudice) for specific SGM subgroups (e.g., gender minority people, SGM BIPOC), further research using validated measures to examine the impact of multiple types of oppression-based stressors on alcohol outcomes is needed. Future research should also consider varying contexts in which oppression is inflicted, such as online versus in-person oppression, vicarious versus direct oppression, oppression occurring outside versus within the SGM community, the type of perpetrator(s), or whether oppression is experienced individually or within a romantic couple or group. Finally, although there has been an increase in longitudinal and experience sampling studies of oppression-based stressors and alcohol use among sexual minority populations, the literature examining the associations between cissexism and alcohol use among gender minority people and intersectional oppression-based stressors and alcohol use among SGM BIPOC is lagging far behind.

One strength, but also a limitation of this review, is that the intersectional lens applied throughout primarily covered only the intersections of gender identity, sexual orientation, and race/ethnicity and their related oppressions. Research is needed on other intersections of identities and oppression (e.g., sexism, classism, ableism, ageism). Qualitative methods provide a rich and in-depth understanding of the tenets of intersectionality theory; nonetheless, researchers can review several suggestions regarding the use of quantitative methods in intersectional research.^215^–^217^

Additional research is needed to identify moderating and mediating mechanisms that help refine our understanding of the link between oppression-based stressors and alcohol use. Given that most of this literature has been cross-sectional,^4^,^5^,^40^,^147^ researchers need to examine these mechanisms using more sophisticated methodologies and analytic strategies^147^ as well as identify novel culturally and developmentally relevant mechanisms across the lifespan. Beyond individual-level factors, mechanisms at the structural level have been entirely understudied and need further investigation. In addition to the continued inquiry into oppression-based stress, motivational and positive reinforcement models (e.g., drinking to celebrate, be sexual, connect with others) should be examined to provide a comprehensive understanding of mechanisms driving drinking among SGM people. Similarly, well-known factors that impact drinking and AUD among the broader population (e.g., stressors and traumatic experiences unrelated to oppression; genetic factors such as family history of AUD), should be also considered. Research to understand how oppression shapes and interacts with sociocultural and psychophysiological mechanisms (e.g., social norms, allostatic load) to influence drinking may also help shed light on inconsistent results in the literature.

Increased rigor in the application of research methods and addiction theories that are commonly used in the wider alcohol literature is needed in the SGM alcohol literature. First, as most of the literature base has focused on primary alcohol outcomes (i.e., quantity and frequency of alcohol use), additional alcohol outcomes should be considered (e.g., blackouts, craving, alcohol cues, withdrawal, intoxication, AUD severity) using established measures and paradigms (e.g., cue reactivity, alcohol demand tasks). Second, craving is a hallmark feature of AUD, yet limited work has examined the impact of oppression-based stressors on craving in the SGM literature.^133^,^186^,^187^ Heterosexism-based stress may elicit alcohol craving, and alcohol cues may maintain or potentiate craving^133^ and lead to alcohol consumption. Therefore, future work should incorporate the context of drinking and related cues. Third, with the exception of three studies,^180^,^182^,^188^ there is a lack of addiction theory-driven investigation of how the progression along the addiction continuum^155^,^218^ may impact the link between etiological factors (e.g., oppression-based stress) and alcohol use outcomes. Future research needs to consider addiction severity, which could help better explain mixed or inconsistent findings in the literature related to stress and drinking among SGM. Relatedly, research is needed to examine the impact of oppression-based stressors on the initiation and progression of alcohol use and the development of AUD over time. Fourth, studies have conflated sex and gender when focusing on alcohol outcomes as well as used inconsistent definitions of hazardous drinking.^3^ As such, researchers can improve the rigor of alcohol research among gender minority populations by following recommendations for the use of the AUDIT with gender minority populations^219^ and making alcohol measures more gender inclusive.^220^

### Implications for Resilience Factors and Interventions

Oppression-based stressors exist in every domain in SGM people’s lives and can impact their alcohol use; thus, protective, resilience, and resistance factors and interventions also must be identified and applied in every domain. At the structural level, affirming laws and policies that protect and bolster the civil rights and humanity of SGM people and eliminate oppression are paramount for addressing alcohol inequities.^207^–^209^ Affirming institutional factors that reduce stigma, create safety and community, raise critical consciousness, and celebrate and empower SGM people are urgently needed.^211^–^213^ The evidence shows that these structural interventions are especially helpful for SGM BIPOC^208^ and can potentially increase the efficacy of alcohol control-related public health policies.^209^ Furthermore, SGM-serving organizations and events must carefully consider the role of sponsorship and promotional efforts by alcohol companies and should engage in health-promoting and hazardous drinking prevention strategies.^119^ Additionally, SGM people are more likely than heterosexual and cisgender individuals to seek treatment for AUD, but they experience more barriers to care and inadequate culturally-sensitive care.^221^–^224^ Thus, more work is needed to intervene with structural treatment barriers, integrate SGM-affirmative and intersectional approaches, and improve cultural humility in care. These interventions must address AUD-specific stigma^225^ and how it impacts the recovery process. Interventions are also needed to disrupt structural barriers that impact social determinants of health (e.g., economic, social).

Strengths-based models acknowledge individual and collective strengths that exist within SGM populations.^226^–^228^ These models underscore strength and resilience factors such as the role of social support and community in promoting wellbeing in SGM populations;^226^–^228^ thus, interventions that promote social connection and community should be prioritized. While promoting community connection and support is vital, it is important to note that prior work has yielded mixed findings, with community connection being both a protective and a risk factor for alcohol use.^2^,^146^ This may be in part due to the aforementioned cultural and permissive drinking norms and exploitation of SGM communities by the alcohol industry. SGM individuals may also drink to connect, celebrate, and be in community with other SGM people. Additionally, the SGM community is not a monolith, and different subgroups can experience unique types of oppression-based stress within the SGM community (e.g., anti-bisexual prejudice, cissexism, racism, sexism, xenophobia, classism, ageism, ableism, religion-based oppression); thus, community interventions must consider these nuances and intersectional experiences.

Individual-level interventions and treatments that affirm SGM individuals and promote their coping skills and resistance to oppression and its related deleterious affective, cognitive, and interpersonal effects are needed to address hazardous drinking and AUD. Existing interventions for SGM populations have primarily focused on sexual minority men,^229^,^230^ and there is a significant lack of interventions for sexual minority women and gender minority people.^4^,^229^–^231^ Therapies that affirm sexual orientation, such as an affirming transdiagnostic cognitive-behavioral therapy and affirming counseling, show some efficacy in reducing alcohol use among young sexual minority men^232^ and sexual minority women.^4^,^229^–^231^ Gender-affirming interventions are needed, including ensuring access to gender-affirming care (e.g., affirming medical interventions, such as gender-affirming hormones and surgeries), because these interventions are protective against HED and AUD in gender minority people.^233^,^234^ Given that oppression-based stress is a traumatizing process and that SGM groups experience a high prevalence of violence, future interventions should also be trauma-informed and sensitive to the multiple contexts in which victimization may be experienced (e.g., intimate relationships).^235^ While social norms interventions (e.g., personalized normative feedback) have been efficacious in reducing alcohol use more generally, there is a paucity of intervention research that has tested their efficacy with SGM populations. Existing studies have yielded promising results in reducing alcohol use among sexual minority women^236^ and sexual minority men.^237^ There is also a need to adapt existing empirically supported alcohol interventions to consider the unique cultural factors and needs of SGM populations.^229^–^231^

Future interventions also must integrate intersectional and social justice approaches to reduce and ultimately eliminate alcohol inequities among SGM people. For example, the radical healing framework was developed to promote the well-being and liberation of BIPOC communities and focuses on the promotion of critical consciousness, hope, resistance, authenticity, and community.^238^ Moreover, strengths-based models underscore the role of identity pride and esteem, emotional awareness, and a future orientation that incorporates hope and optimism.^226^,^227^ Although these models are significantly underutilized in the SGM alcohol field, they are excellent future directions for SGM populations.
